# Web-Based Platform for the Chilean Cardiac Surgery Registry: Algorithm Development and Validation Study

**DOI:** 10.2196/70147

**Published:** 2025-11-11

**Authors:** Sergio Guinez-Molinos, Enrique Seguel, Jaime Gonzalez, Benjamin Castillo

**Affiliations:** 1 Simulation and Biomedical Informatics Laboratory, Faculty of Medicine, Universidad de Talca, San Miguel 3748, PO Box 721, Talca, 3460000, Chile, +56996195268; 2 Cardiovascular Centre, Guillermo Grant Benavente Hospital, Concepción, Chile; 3 Department of Surgery, Faculty of Medicine, University of Concepción, Concepción, Chile; 4 Centro de Investigación de Estudios Avanzados del Maule (CIEAM), Vicerrectoría de Investigación y Postgrado, Universidad Católica del Maule, Talca, Chile

**Keywords:** cardiac surgery, health informatics, clinical registry, EuroSCORE II, Chile

## Abstract

**Background:**

Cardiac surgeries in Chile lack a national registry for systematic data collection and analysis, limiting insights into procedural outcomes and patient demographics. In response to this gap, we developed a web-based platform to support the documentation of high-complexity cardiac surgeries.

**Objective:**

This study aimed to design, develop, and implement a cardiac surgery data collection and analysis platform that conforms to international standards to support clinical decision-making and research initiatives.

**Methods:**

A web-based platform was developed using the model-view-controller architecture, incorporating input from health care professionals and based on the fourth European Association for Cardio-Thoracic Surgery adult cardiac surgical database report. The platform captures more than 160 clinical variables across 15 categories, spanning preoperative, intraoperative, and postoperative stages.

**Results:**

The most significant outcome of this study is the development of the first online platform for documenting cardiac surgeries in Chile. Since its implementation in 2014, the platform has documented more than 4800 cardiac surgeries, establishing it as the largest database for a single institution in Latin America. The platform offers real-time access to data, supports planning and resource allocation, and enables the systematic evaluation of clinical outcomes. Integrating the European System for Cardiac Operative Risk Evaluation II risk model enables a standardized assessment of mortality risk.

**Conclusions:**

The platform contributes to the collection of cardiac surgery data in Chile, enabling evidence-based clinical decision-making and informed public health planning. It has documented cardiac surgeries for 10 years and has become the official registry tool for cardiac surgeries. By 2026, its application will be extended to 2 more centers, with the expectation that it will soon become the national database of cardiac surgeries. Future developments should improve scalability, interoperability, and data analysis to establish a national registry and further align Chilean cardiac surgery practices with international standards.

## Introduction

Integrating health information systems into clinical practice has transformed cardiac surgery, establishing a basis for evidence-based decision-making [1]. Electronic medical records, easier access to clinical data, and statistical analysis tools have improved the approaches to treating and managing various cardiac conditions [2].

International scientific societies have recognized the importance of standardized data collection and have developed electronic databases to collect and analyze cardiac surgery outcomes. The Society of Thoracic Surgeons database in the United States has become the largest cardiac surgery registry in the world [3]. Other initiatives include the databases created by the Spanish Society of Thoracic and Cardiovascular Surgery [4], the German Society of Thoracic and Cardiovascular Surgery [5], the British Society of Cardio-Thoracic Surgeons [6], the Australian and New Zealand Society of Cardio-Thoracic Surgeons [7], and the Japanese Society of Cardiovascular Surgery [8]. In Europe, the European Association for Cardio-Thoracic Surgery (EACTS) [9,10], with the support of the Society of Thoracic Surgeons, has also made significant contributions to the worldwide network of cardiac surgery registries. These databases highlight the importance of systematic registries in understanding the epidemiology and outcomes of cardiac surgery [11].

In Chile, there is no national epidemiological registry or health information system that systematically gathers data on cardiac surgery and related procedures [11]. As a result, the actual number of procedures performed, their short- and long-term outcomes, and the demographic and clinical profiles of patients remain largely unknown. This lack of structured data hampers the objective evaluation of health interventions, limits follow-up efforts, and restricts the development of evidence-based public health policies. Consequently, decision-making in this field often becomes reactive, lacking the necessary information to assess impact or allocate resources efficiently and accurately.

To address these challenges, the first web-based cardiac surgery registry platform in Chile has been developed and implemented. Specifically designed to document highly complex cardiac procedures, this initiative emerged from the joint efforts of the Cardiac Surgery Service at Guillermo Grant Benavente Hospital, the Department of Surgery at the University of Concepción, and the Center for Simulation and Biomedical Informatics at the Faculty of Medicine of the University of Talca. The primary aim of this platform is to bridge the current data gap and establish a robust foundation for enhancing surgical outcomes in cardiac care nationwide.

Currently, Guillermo Grant Benavente Hospital is the only center fully integrated into this electronic registry. In parallel, formal discussions with the Ministry of Health are underway, with a long-term vision of scaling this initiative into a nationwide platform that can serve as the cornerstone for monitoring quality, benchmarking outcomes, and guiding clinical decision-making in Chilean cardiac surgery.

## Methods

A standard software engineering methodology was used to develop a platform for recording cardiac surgery and procedure data [12]. Close collaboration with cardiology experts was essential from the outset, with nurses and physicians playing a key role in the iterative requirements-gathering process. The platform’s scope, modules, and sections were defined through a series of meetings at the Cardiac Surgery Registry (Table 1). This collaborative approach, involving different clinical and IT profiles such as cardiac surgeons, cardiologists, perfusionists, nurses, and biomedical informatics specialists, resulted in a detailed technical document that served as the basis for the software’s development.

**Table 1.** Summary of the requirements survey, with the main functional and nonfunctional requirements to be covered by the platform.

Modules	Requirements	Detailed requirements
Platform administration	Functional	The system must implement user authorization and authentication. User accounts must have profiles within the platform according to their roles in the medical process registry. Each role should be associated with specific privileges or functionalities. The platform should provide functionalities to create, update, and delete user accounts, manage hospital lists, and refer health care services. Users can have different roles: administrator, professional, or data logger.
Cardiac surgery registry	Functional	The system should be capable of storing data related to complex cardiac surgeries and procedures. The available input fields should be based on the EACTS^a^ dataset modified to suit local requirements. The system should also automatically calculate indicators such as EuroSCORE II^b^ to support decision-making processes. Multilanguage (English and Spanish).
Patients, institutions, and inventory	Functional	The system should securely store patient data, ensuring anonymity, and avoiding records. It should also store data related to personnel, institutions, and equipment used during interventions and patient monitoring. The recorded data must be interoperable, allowing it to be shared with other platforms in the ecosystem.
Reporting and data recovery	Functional	The system must allow the export of data related to the procedures. It must be able to generate reports and visualizations of the data recorded and the use of the platform. Data retrieval and validation must be guaranteed.
All modules	Nonfunctional	Ease of use and simplicity are essential, especially for data entry associated with surgeries or medical procedures and visualization. Data security must be ensured with strong authorization, authentication, and privacy measures. Fail-safe mechanisms must be implemented to ensure the accuracy of data capture and prevent data loss or inconsistencies. The platform must support scalability and data independence, allowing the addition of new data categories without affecting existing data or data capture processes. It must also facilitate seamless integration with other platforms.

a EACTS: European Association for Cardio-Thoracic Surgery.

b EuroSCORE II: European System for Cardiac Operative Risk Evaluation.

The analysis of clinical requirements highlighted 2 essential elements for the platform’s design and development. First, the EACTS guidelines should be followed [13], considering the clinical variables recorded in that database and adapting the registry to local needs. Second, the platform should incorporate automatic calculation of the European System for Cardiac Operative Risk Evaluation II (EuroSCORE II) mortality risk indicator to support clinical decision-making processes [10,14].

### Functional and Nonfunctional Requirements

The cardiac surgery registry is designed for health care professionals to record and query data related to medical procedures. Administrators manage the platform’s maintenance, including user privileges and master tables. Both cardiac surgeons and nurses who are part of the clinical procedural team can enter data related to complex surgeries and procedures. Surgeons are associated with specific guidelines and can review the corresponding records from anywhere with an internet connection.

A fundamental aspect is the security of sensitive data. A data security layer is implemented that guarantees, in accordance with national data protection legislation, the appropriate authorization, authentication, and privacy of records.

The platform features multilevel user authentication, encrypted connections (secure sockets layer and transport layer security) [15], anonymization of patient identifiers, and storage on secure servers with regular backups. Access is based on roles (surgeons, nurses, and administrators) and is designed to restrict unauthorized access to sensitive information.

### Cardiac Surgery Clinical Sections

**Figure 1.**
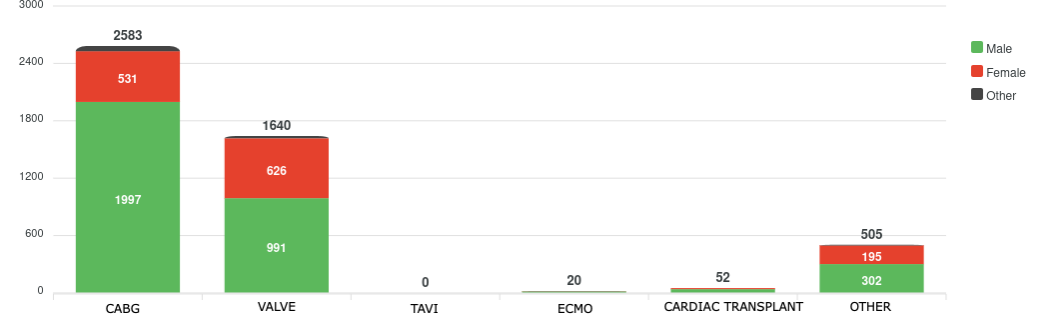
Chart illustrating the distribution of procedures by type according to gender. CABG: coronary artery bypass grafting; ECMO: extracorporeal membrane oxygenation; TAVI: transcatheter aortic valve implantation.

The types of cardiac surgeries included in the registry are as follows: coronary artery bypass grafting (CABG), valve surgeries (aortic, mitral, and tricuspid), and combined CABG and valve procedures (Figure 1). In addition, extracorporeal membrane oxygenation and heart transplants are being implemented on a pilot basis.

According to the surgeons’ and their teams’ requirements, clinical sections were established for data collection on cardiovascular surgeries. Key categories included patient demographics, cardiovascular history, previous interventions, preoperative risk factors, hemodynamics, and immediate status before surgery. Detailed information on the procedure, echocardiogram findings, and myocardial protective measures was also documented, ensuring a thorough understanding of the surgical and intraoperative contexts. Postoperative complications, discharge outcomes, and long-term patient follow-up were systematically tracked to assess recovery and survival rates. All the above are based on the EACTS structure [10], from which we derived our clinical sections (Table 2).

**Table 2.** The platform will represent the main clinical sections.

Clinical section	Description
Hospitalization details	Captures patient identification, admission specifics, health care service, and the urgency of intervention
Cardiovascular history	Documents heart-related conditions such as angina, myocardial infarctions, and congestive heart failure
Previous interventions	Records prior surgeries, angioplasties, and their dates
Preoperative risk factors	Assesses risks, including weight, smoking history, and preexisting medical conditions
Preoperative hemodynamics and catheterization	Includes diagnostic metrics such as coronary vessel status and ejection fraction
Preoperative status and support	Notes presurgery interventions such as IV medications and mechanical support
Operation details	Provides surgical specifics, including type, urgency, and personnel involved
Coronary surgery	If a coronary surgery was performed, it is registered here
Valve surgery	Stenosis, insufficiency, explant type, and other data are registered for valve surgery (aortic, mitral, tricuspid, and pulmonary)
Echocardiogram	Records imaging results, focusing on valve conditions and ventricular measurements
Other procedures	Other cardiac and noncardiac procedures relevant to the operation are recorded here
Perfusion and myocardial protection	Describes intraoperative techniques to protect the heart
Postoperative complications	Tracks complications such as reoperations and system failures
Discharge details	Summarizes outcomes, including discharge status or causes of death
Patient monitoring	Documents postdischarge events to evaluate long-term outcomes and mortality

The clinical sections with all their variables are important for patient follow-up. Follow-up is standardized at discharge, 30 days after surgery, and 1 year after surgery. Additional follow-up points are added if adverse events occur.

Responsibility for data entry is defined a priori. Data will be entered primarily by surgical nurses and residents during the perioperative care period. Surgeons validate and sign off on each procedure. A registry coordinator (specialist nurse) supervises to ensure completeness. The role of records coordinator is responsible for monitoring the quality of the data entered and the completeness of the record.

By integrating data from all these categories, the study provides valuable insights into factors influencing surgical outcomes, thereby enhancing our understanding of patient risk profiles and the efficacy of interventions (Multimedia Appendix 1). Minimum dataset for the EACTS-based cardiac surgery registry.

### Software Architecture Design

**Figure 2.**
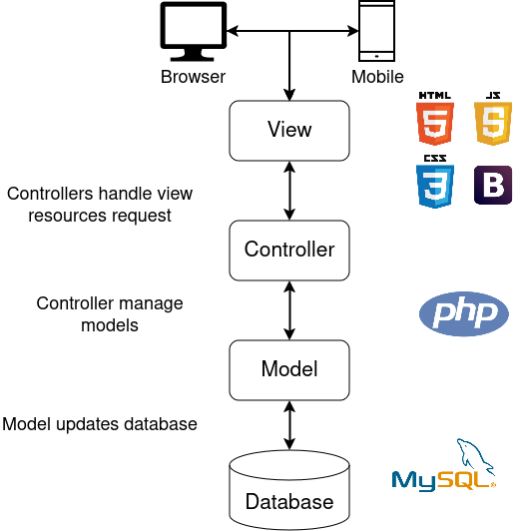
Model-View-Controller architecture for the cardiac surgery platform.

A web platform based on model-view-controller architecture was developed to ensure modularity, scalability, and ease of maintenance [16]. MySQL 5.6 [17] was used for data persistence, and PHP 5.4 [18] was used to implement the controller and model layers, facilitating efficient server-side scripting and database interaction [19]. The display layer was built using HTML, CSS, JavaScript, and Bootstrap 3, ensuring a dynamic and user-friendly interface with an adaptive design for accessibility across various devices (Figure 2) [20].

The development process began with the design of the database model, followed by the implementation of the server-side components, including security protocols. Responsiveness and usability were prioritized in the client interface. After initial development, a 6-month beta testing phase was conducted at the Guillermo Grant Benavente Hospital in Concepción, a leading cardiology center in Chile. The results of this phase served as a guide to refine the platform and prepare it for production deployment.

### Hospital Inclusion Criteria

The platform is currently being used at Guillermo Grant Benavente Hospital in Concepción, Chile. To increase coverage and include more centers, the following inclusion criteria must be satisfied.

- Perform high-complexity cardiac surgeries.
- Have dedicated surgical teams with stable caseloads.
- Commit to institutional agreements ensuring data quality, anonymization, and adherence to ethical and legal requirements.

### Ethical Considerations

The project and use of the registry were reviewed and approved by the Scientific Ethics Committee of the Faculty of Medicine at Universidad de Concepción (CEC 16/2024). For the purpose of this paper, all data were anonymized.

## Results

### Overview

**Figure 3.**
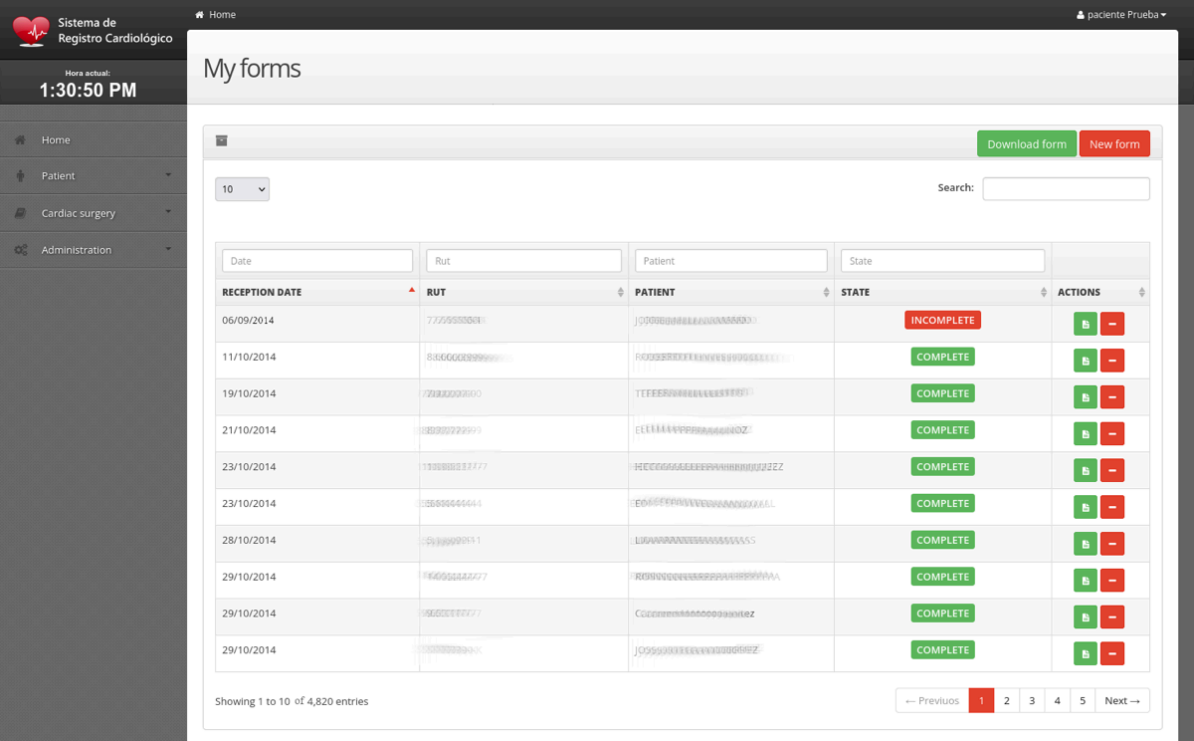
Screenshot of the cardiac surgery registry section. The platform facilitates the registration and management of cardiac surgeries performed on patients from various locations in south-central Chile.

We created a web platform for systematically recording clinical data from adult cardiac surgeries at the main hospital in south-central Chile (Figure 3). The platform enables efficient data storage, management, querying, and visualization. Since its launch in 2014, it has become the most important registry of complex cardiac procedures in Chile and Latin America [14], documenting more than 4800 surgeries in the past decade (Figure 3), considering 54% (2592/4800) CABG, 34% (1632/4800) valve surgeries, and 12% (576/4800) other complex procedures.

The platform is accessible from anywhere with an internet connection and is compatible with various devices, including desktop computers and mobile devices. Its bilingual interface, available in both Spanish and English, ensures accessibility to a wide range of users. Aggregated datasets for advanced analysis, supporting clinical operations. A module dedicated to “Statistics and Graphs” provides predefined reports for immediate use (Figure 1). At the same time, an integrated export tool allows users to extract data.

**Figure 4.**
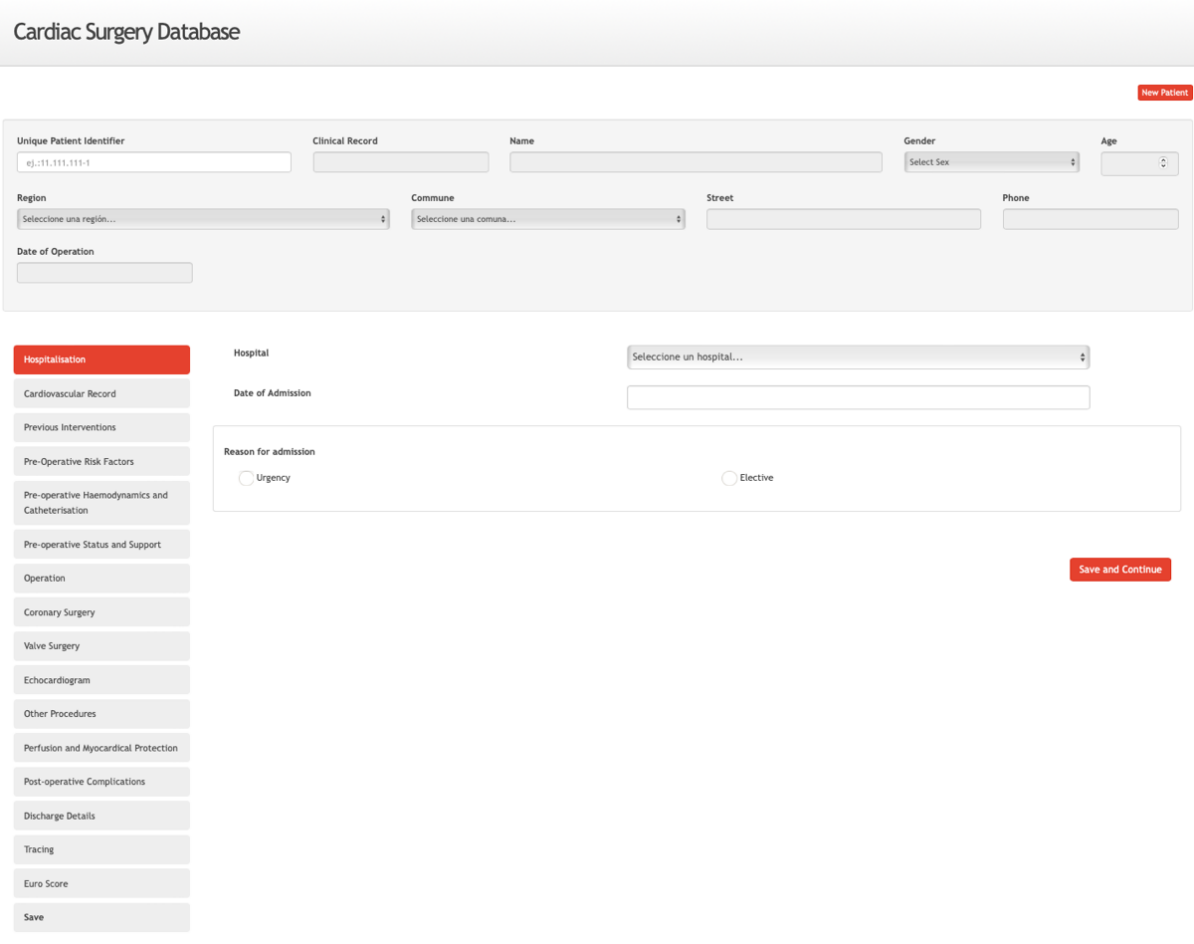
Screenshot of information recording sections from any internet-connected device.

The platform is designed to capture more than 160 structured data points spanning preoperative, intraoperative, and postoperative stages, categorized into 15 sections for comprehensive data collection and streamlined analysis (Figure 4). These sections include patient demographics, preoperative conditions, risk factors, procedural details, postoperative outcomes, discharge information, and follow-up data (Table 2 and Multimedia Appendix 1).

The preoperative stage begins with the hospitalization section, which records patient identifiers and procedural details. The cardiovascular history section captures previous conditions, such as angina and heart failure, while the previous interventions section documents earlier angioplasties and surgeries. Sections on risk factors, hemodynamics, and catheterization status support and detail comorbidities, clinical status, and supportive measures.

The intraoperative section records details of the intervention, including the participating professionals, the reasons for surgery, and the types of procedures performed. Specialized sections for coronary, valve, and echocardiograms provide specific insights, while additional sections cover cardiac and noncardiac procedures, as well as myocardial protection.

The postoperative complications section captures postoperative data, discharge details, patient monitoring, and follow-ups. This structured design ensures standardized and detailed data collection, supporting clinical operations and research initiatives.

### The EuroSCORE II Module

The EuroSCORE II is a risk prediction model used to estimate the probability of mortality in patients undergoing cardiac surgery [21]. It was developed as an updated version of the original EuroSCORE to reflect contemporary clinical practices and improve accuracy. Widely used in clinical and research settings, EuroSCORE II helps health care professionals assess surgical risks, guide decision-making, and benchmark institutional performance [9,21].

**Figure 5.**
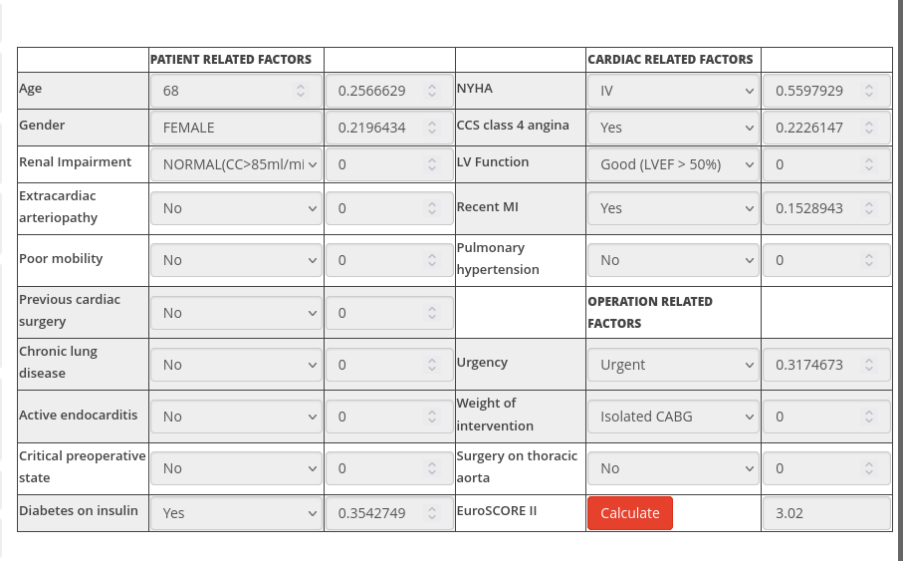
Screenshot of the European System for Cardiac Operative Risk Evaluation II module that emphasizes how each factor contributes to the total risk score. CABG: coronary artery bypass grafting; CCS: Canadian Cardiovascular Society; EuroSCORE II: European System for Cardiac Operative Risk Evaluation; LV: left ventricle; LVEF: left ventricular ejection fraction, MI: myocardial infarction; NYHA: New York Heart Association.

The indicator is derived from factors grouped into 3 main categories: patient-related factors, cardiac-related factors, and operation-related factors (Figure 5). Each factor is assigned a predefined score based on acceptable values, which contributes to the overall risk assessment.

To determine the final EuroSCORE II value, all individual scores are summed and applied to a logistic function, which adjusts the raw score into a risk percentage. This process provides an accurate and standardized surgical risk assessment, aiding clinical decision-making and patient counseling.

Each factor is assigned a predefined weight or coefficient based on its relative impact on mortality risk, as determined through large-scale statistical analysis. For example, age contributes progressively higher weights as it increases beyond 60 years, and pre-existing conditions such as renal dysfunction or severe comorbidities significantly increase the risk score.

The cumulative score is then calculated by summing the weighted contributions of all factors. This total score is applied to a logistic regression formula to translate it into a percentage probability of mortality:


$$ES\;II\;\%=\frac{e^{\beta_{0}+\beta_{1}x_{1}+\beta_{2}x_{2}+\ldots +\beta_{n}x_{n}}}{1+e^{\beta_{0}+\beta_{1}x_{1}+\beta_{2}x_{2}+\ldots +\beta_{n}x_{n}}}\times 100\%$$

Where:

- e is the base of the natural logarithm
- $\beta_{0}$ is the intercept term.
- $\beta_{1},\beta_{2},\beta_{3}\ldots .\beta_{n}$ are the regression coefficients for each factor.
- $x_{1},x_{2},x_{3}\ldots .x_{n}$ are the weighted values of the patient’s factors (Figure 5).

## Discussion

### Principal Findings

We present a proposal for the systematic registration and analysis of cardiac surgeries in Chile, aiming to collect clinical data and stratify risk. To address this, we developed a web platform tailored to local needs, offering an intuitive and user-friendly tool. In addition, the platform’s alignment with international standards, such as the EACTS guidelines, and the integration of the EuroSCORE II risk model underscore its potential to support both clinical and research applications.

Currently, the registry is implemented and actively used at Guillermo Grant Benavente Hospital, one of the largest reference centers in Chile. Nevertheless, formal agreements are underway with 2 additional cardiac surgery centers (Hospital San Juan de Dios de Curicó and Hospital Clínico Regional de Antofagasta) to expand its use starting in 2025. The long-term vision, discussed with the Ministry of Health, is to scale it as a national platform.

### Platform Implementation and Usability

The platform successfully implemented more than 160 data points, structured across 15 clinical sections, distributed among preoperative, intraoperative, and postoperative variables. Its modular and scalable architecture facilitated integration into clinical workflows while ensuring accessibility through a bilingual interface compatible with various devices. These features have been instrumental in promoting its adoption and ease of use by health care professionals in multiple settings.

### Future Work for the Platform and Risk Assessment

Future development should incorporate artificial intelligence algorithms and advanced statistical methodologies to enhance the platform’s impact and improve the accuracy of current risk models [22]. These approaches could improve the predictive accuracy of risk assessments by accommodating complex, nonlinear relationships between variables. In addition, expanding the platform’s interoperability with other health information systems would facilitate broader data sharing and benchmarking, thereby aligning local practices with international standards. Additional efforts should be made to engage users in continuous feedback loops to refine the platform’s functionality and usability.

The implementation of this platform helps address the challenges associated with fragmented data and limited risk stratification capabilities in Chile. With 4800 records, it is currently the most significant database (considering only 1 center) in Chile and Latin America [1,14].

The collection of data and calculation of the EuroSCORE II will allow the validation of this risk scale in a Latin American population. The differences that can be observed would eventually allow this scale to be adjusted or calibrated to this population. Using a risk scale adjusted to the population to which it is applied will enable better clinical decision-making for our patients.

Based on recent discussions with the Chilean Ministry of Health, it can be extended to the rest of the country, enabling standardized and centralized data collection and laying the foundation for evidence-based improvements in surgical quality and patient safety [2,11]. In addition, risk models such as EuroSCORE II should be recalibrated for the Chilean population to provide accurate and actionable information for informed clinical decision-making.

### Conclusions

This study presents the first Chilean web-based platform for collecting cardiac surgery data, addressing the need for systematic documentation of highly complex procedures. The platform has registered more than 4800 surgeries, encompassing 160 clinical variables. This registry aims to support detailed data analysis and improve surgical planning, resource allocation, and risk assessment by integrating the EuroSCORE II module.

The proposed registry platform is a substantial contribution to clinical centers, and future efforts should focus on improving interoperability and integrating advanced analytics to enable scalability on a national scale.

This initiative demonstrates the potential of biomedical informatics, particularly electronic registry systems, to improve health outcomes, align with international standards, and inform evidence-based public health policies in Chile.

## Supplementary material

10.2196/70147 Multimedia Appendix 1 Minimal dataset for record cardiac surgeries based on European Association for Cardio-Thoracic Surgery.

## Abbreviations

CABG: coronary artery bypass grafting
EACTS: European Association for Cardio-Thoracic Surgery
EuroSCORE: European System for Cardiac Operative Risk Evaluation
