# Supplementary material for: Web-Based Platform for the Chilean Cardiac Surgery Registry: Algorithm Development and Validation Study
Source: JMIR Cardio. 2025 Nov 11;9:e70147. doi: 10.2196/70147 (PMC12604825; doi:10.2196/70147)
Supplement: Multimedia Appendix 1 [file cardio-v9-e70147-s001.docx]

**Table S1.**

| **Section** | **Description** | **Variables** |
| --- | --- | --- |
| Hospitalization | Patient identification information such as unique identifiers, demographic details (age, gender, and address), and contact information were recorded. Hospitalization specifics, including the healthcare service, hospital name, admission date, and the primary reason for admission, were also documented to establish the clinical context. | - RUT - Patients register number - Patient name - Gender - Age - Address - Phone - Hospital - Healthcare service - Date of admission - Reason for admission |
| Cardiovascular history | A detailed record of the patient’s heart-related medical history was maintained, including angina, functional capacity, previous myocardial infarctions, and their characteristics. Additionally, data on congestive heart failure episodes was included to provide a baseline for cardiovascular assessment. | - Angina - Functional capacity - Number of previous myocardial infractions - The most recent myocardial infraction - Congestive heart failure |
| Previous interventions | Data was gathered regarding earlier medical procedures, including prior angioplasties and cardiac surgeries. Specifics such as the number and dates of previous interventions were noted to assess the surgical history and its potential impact on outcomes. | - Previous angioplasty - Number of previous heart operations - Date of last angioplasty - Date of last cardiac surgery - Previous cardiac, vascular, or thoracic surgery |
| Preoperative risk factors | Patient risk factors were identified, including weight, height, smoking history, and co-morbidities such as diabetes, hypertension, nephropathy, and chronic lung disease. Pre-existing conditions like cerebrovascular disease and extra-cardiac arteriopathy were also documented to evaluate risk profiles comprehensively. | - Weight - Height - Smoking history - Smoking history- packs/year - Diabetes - Hypertension - Hypercholesterolemia - Nephropathy - Last preoperative creatinine - Chronic lung disease - Extra-cardiac arteriopathy - Cerebrovascular disease type - Neurological dysfunction - Carotid bruits - Preoperative heart rhythm |
| Preoperative hemodynamics and catheterization | Diagnostic data from catheterization procedures were collected, covering coronary vessel conditions, left main stem disease, and vital hemodynamic metrics such as ejection fraction and pulmonary pressures. This information was critical for preoperative planning. | - Left or right heart catheterization - Date of last catheterization - Number of diseased coronary vessels - Left main stem disease - Ejection fraction - Ejection fraction value (or unmeasured) - PA systolic (or unmeasured) - AV gradient (or unmeasured) - LVEDP (or unmeasured) - Mean PAWP/LA (or unmeasured) |
| Preoperative status and support | The patient’s condition immediately before surgery was documented, including the use of intravenous medications, mechanical ventilation, and circulatory support devices. The presence of cardiogenic shock was also noted. | - IV nitrates - IV inotropes - Mechanical ventilation - Cardiogenic ventilation - Cardiogenic shock - Balloon contra pulsation intra-aortic |
| Operation | Procedural data encompassed the surgery type, urgency, and team members involved, such as surgeons and anesthetists. Specifics of coronary and valve surgeries were documented, including valve stenosis, insufficiency, and implant type. | - Date of operation - Surgeons (1-3) - Anesthetist - Perfusionist - Operative urgency - The main reason for the urgency - Procedure types - Other cardiac procedure - Other non-cardiac procedure |
| Coronary surgery | If a coronary surgery was performed, it is registered here. | - Number of distal arterial anastomosis - Number of distal venous anastomosis - Arteries used as grafts |
| Valve surgery | Stenosis, insufficiency, type of explant, and other data are registered for valve surgery (aortic, mitral, tricuspid, and pulmonary) | - Type of valve - Stenosis - Insufficiency - Explant type - Reason for repeat valve surgery - Valve procedure - Implant type - Infectious endocarditis - Model/mark (implant) - Valve/ring size (implant) - Native valve pathology |
| Echocardiogram | Diagnostic imaging results were analyzed to evaluate valve conditions and ventricular dimensions. Measurements such as gradients and diameters and information on endocarditis or cardiac tumors were included. | - Date - Left ventricle – diastolic diameter (mm) or unmeasured - Left ventricle – systolic diameter (mm) or unmeasured - Left ventricle -Ejection fraction (mm) or unmeasured - Aortic valve – Maximum gradient (mmHg) or unmeasured - Aortic valve – Medium gradient (mmHg) or unmeasured - Aortic valve – Maximum speed (mm/s) or unmeasured - Aortic valve – Area (mm/s) or unmeasured - Aortic valve – insufficiency - Mitral valve – Maximum gradient (mmHg) or unmeasured - Mitral valve – Medium gradient (mmHg) or unmeasured - Mitral valve – Maximum speed (mm/s) or unmeasured - Mitral valve – Area (mm/s) or unmeasured - Mitral valve – insufficiency - Tricuspid valve – insufficiency - PSAP (mmHg) or unmeasured - Right cavities - Aortic root (mm) or unmeasured - Left atrium (mm) or unmeasured - Endocarditis - Endocarditis – valve - Endocarditis – vegetation - Endocarditis – Abscess - Endocarditis - Prosthesis detachment - Endocarditis – Cardiac tumor - Endocarditis – other - Diagnosis |
| Other procedures | Other cardiac and non-cardiac procedures relevant to the operation are recorded here. | - Other cardiac procedures detail - Other non-cardiac procedures detail - Segments of aorta - Aortic procedure - Endoscopic vein |
| Perfusion and myocardial protection | Intraoperative measures to protect the myocardium were described, including cardioplegia techniques, extracorporeal circulation time, and methods of circulatory assistance. | - Extracorporeal circulation - Arterial cannulation - Venous cannulation - Perfusion temperature - Myocardial protection - Cardioplegia- solution - Cardioplegia- temperature - Cardioplegia- infusion mode - Cardioplegia- timing - Balloon contra-pulsation - Reason for balloon contra-pulsation use - Extracorporeal circulation time - Cumulative cross-clamp time - Total circulatory arrest time - Circulatory assistance |
| Post-operative complications | A comprehensive record of complications, such as re-operations, neurological events, or multisystem failure, was maintained to monitor immediate outcomes. | - Date - Re-operation - New post-operative stroke - New post-operative dialysis - Multisystem failure - Preoperative infraction |
| Discharge details | Patient discharge outcomes were documented, including the date, destination, and status. For deceased patients, the principal cause of death was identified. | - Date of discharge or death - Destination on discharge - Patient status at discharge - Principal cause of death |
| Patient monitoring | Post-discharge events, including survival status updates and mortality causes, were tracked to analyze long-term outcomes and calculate relevant indicators. | - Status - Date of death - Cause of death |

**References**

1. The European association for cardio-thoracic surgery. EACTS Adult Cardiac Database File Specification. 2018 Mar.

2. Head SJ, Howell NJ, Osnabrugge RLJ, Bridgewater B, Keogh BE, Kinsman R, Walton P, Gummert JF, Pagano D, Kappetein AP. The European association for cardio-thoracic surgery (EACTS)database: An introduction. European Journal of Cardio-thoracic Surgery 2013;44(3):175–181. doi: 10.1093/ejcts/ezt303
